# Postmortem Neocortical ^3^H-PiB Binding and Levels of Unmodified and Pyroglutamate Aβ in Down Syndrome and Sporadic Alzheimer’s Disease

**DOI:** 10.3389/fnagi.2021.728739

**Published:** 2021-08-13

**Authors:** Violetta N. Pivtoraiko, Tamara Racic, Eric E. Abrahamson, Victor L. Villemagne, Benjamin L. Handen, Ira T. Lott, Elizabeth Head, Milos D. Ikonomovic

**Affiliations:** ^1^Geriatric Research Education and Clinical Center, VA Pittsburgh Healthcare System, Pittsburgh, PA, United States; ^2^Department of Neurology, University of Pittsburgh School of Medicine, Pittsburgh, PA, United States; ^3^Department of Psychiatry, University of Pittsburgh School of Medicine, Pittsburgh, PA, United States; ^4^Department of Neurology, UC Irvine School of Medicine, Orange, CA, United States; ^5^Department of Pathology and Laboratory Medicine, UC Irvine School of Medicine, Orange, CA, United States

**Keywords:** Alzheimer’s disease, amyloid, cerebral amyloid angiopathy, default mode network, Down syndrome, Pittsburgh Compound-B, pyroglutamate

## Abstract

Individuals with Down syndrome (DS) have a genetic predisposition for amyloid-β (Aβ) overproduction and earlier onset of Aβ deposits compared to patients with sporadic late-onset Alzheimer’s disease (AD). Positron emission tomography (PET) with Pittsburgh Compound-B (PiB) detects fibrillar Aβ pathology in living people with DS and AD, but its relationship with heterogeneous Aβ forms aggregated within amyloid deposits is not well understood. We performed quantitative *in vitro* ^3^H-PiB binding assays and enzyme-linked immunosorbent assays of fibrillar (insoluble) unmodified Aβ40 and Aβ42 forms and *N*-terminus truncated and pyroglutamate-modified AβNpE3-40 and AβNpE3-42 forms in postmortem frontal cortex and precuneus samples from 18 DS cases aged 43–63 years and 17 late-onset AD cases aged 62–99 years. Both diagnostic groups had frequent neocortical neuritic plaques, while the DS group had more severe vascular amyloid pathology (cerebral amyloid angiopathy, CAA). Compared to the AD group, the DS group had higher levels of Aβ40 and AβNpE3-40, while the two groups did not differ by Aβ42 and AβNpE3-42 levels. This resulted in lower ratios of Aβ42/Aβ40 and AβNpE3-42/AβNpE3-40 in the DS group compared to the AD group. Correlations of Aβ42/Aβ40 and AβNpE3-42/AβNpE3-40 ratios with CAA severity were strong in DS cases and weak in AD cases. Pyroglutamate-modified Aβ levels were lower than unmodified Aβ levels in both diagnostic groups, but within group proportions of both pyroglutamate-modified Aβ forms relative to both unmodified Aβ forms were lower in the DS group but not in the AD group. The two diagnostic groups did not differ by ^3^H-PiB binding levels. These results demonstrate that compared to late-onset AD cases, adult DS individuals with similar severity of neocortical neuritic plaques and greater CAA pathology have a preponderance of both pyroglutamate-modified AβNpE3-40 and unmodified Aβ40 forms. Despite the distinct molecular profile of Aβ forms and greater vascular amyloidosis in DS cases, cortical ^3^H-PiB binding does not distinguish between diagnostic groups that are at an advanced level of amyloid plaque pathology. This underscores the need for the development of CAA-selective PET radiopharmaceuticals to detect and track the progression of cerebral vascular amyloid deposits in relation to Aβ plaques in individuals with DS.

## Introduction

Individuals with Down syndrome (DS) have an overabundance of amyloid-β (Aβ) peptide production due to trisomy of chromosome 21, which harbors the Aβ-precursor protein (APP) gene (Oyama et al., 1994), and they typically develop Alzheimer’s disease (AD) pathology by the fifth decade of life (Davidson et al., 2018). The primary histopathological features of AD that are present in the DS brain include amyloid plaque deposits of fibrillar Aβ peptides and neurofibrillary tangles of over-phosphorylated tau protein (Wisniewski et al., 1985; Mann, 1988; Dickson, 2005; Head et al., 2016; Davidson et al., 2018; Perez et al., 2019). As in AD, fibrillar Aβ also accumulates in the brain vasculature (cerebral amyloid angiopathy, CAA) in the DS brain, at levels exceeding those seen in normal aging (Vinters, 1987; Carmona-Iragui et al., 2017; Head et al., 2017; Davidson et al., 2018).

Recent improvements in medical care have contributed to the increased longevity of individuals with DS, and advancements in diagnostic biomarkers have facilitated studies of key questions regarding the interconnected clinical and neuropathological features that develop with age in DS (Neale et al., 2018; Handen et al., 2020; Head and Ances, 2020; Petersen et al., 2020, 2021; Rafii et al., 2020; Hendrix et al., 2021). Positron emission tomography (PET) studies using Pittsburgh Compound-B (^11^C-PiB) and related amyloid-binding radiopharmaceuticals provide insight into regional distributions and temporal changes in Aβ pathology in living people with AD (Klunk et al., 2004; Rowe et al., 2007; Cohen et al., 2012; Mathis et al., 2017; Villemagne et al., 2021) and this technology is being applied increasingly to studies of individuals with DS (Landt et al., 2011; Handen et al., 2012; Hartley et al., 2014, 2017, 2020; Annus et al., 2016, 2017; Lao et al., 2016, 2017, 2018; Cole et al., 2017; Cohen et al., 2018; Neale et al., 2018; Mak et al., 2019a,b; Mihaila et al., 2019; Tudorascu et al., 2019, 2020; Wilson et al., 2019; Cody et al., 2020; Zammit et al., 2020, 2021). PET imaging of brain Aβ pathology and brain metabolism as well as functional connectivity (fMRI) studies have shown that certain brain regions are more vulnerable than others to pathological changes in AD. Cortical association areas contributing to the core regions of the default mode network (DMN), including the frontal cortex, the precuneus, and the posterior cingulate cortex, show functional impairment and amyloid deposition in early AD stages (Buckner et al., 2005; Jones et al., 2011; Palmqvist et al., 2017), and these brain regions are also affected in adults with DS (Tudorascu et al., 2019; Wilson et al., 2019) suggesting that amyloid pathology affects similar cortical circuits in DS and AD. A recent longitudinal ^11^C-PiB PET study reported slower progression of the frontal cortex and precuneus amyloid pathology in nondemented young adults with DS (mean age 37 years) when compared to nondemented elderly (mean age 73 years; Tudorascu et al., 2019). Thus, there is a need for determining if amyloid PET ligand retention is influenced by regional differences in structural and biochemical characteristics of Aβ pathology in DS compared to aging and AD.

Autopsy studies demonstrated that cyano-PiB, a highly fluorescent derivative of PiB which detects Aβ plaques in histological sections from AD brains (Ikonomovic et al., 2008, 2020), also labels Aβ plaques in postmortem DS brain tissue (LeVine et al., 2017; Abrahamson et al., 2019; Perez et al., 2019). In addition, analyses of *in vitro* binding of ^3^H-PiB to postmortem frontal cortex homogenates showed that in DS individuals higher binding levels were associated with more advanced age (LeVine et al., 2017) and that DS individuals between the ages of 43–63 years had significantly higher binding levels compared to cognitively normal elderly between the ages of 78–92 years and cases with mild-moderate AD between the ages of 77–101 years (Abrahamson et al., 2019). However, the contribution of molecularly heterogeneous Aβ forms (Saido et al., 1996; Roher et al., 2017), and their conformational changes when fibrillized (Schlenzig et al., 2009; Chen et al., 2017; Creekmore et al., 2021), on PiB binding is not well understood. Aβ peptides with the C-terminus ending at amino acid 42 predominate in Aβ plaques (Dickson, 1997), and are believed to be the initially deposited and a principal Aβ form in Aβ plaques in both AD and DS (Jarrett et al., 1993; Iwatsubo et al., 1994, 1995; Saido et al., 1995; Mann and Iwatsubo, 1996; Michno et al., 2019; Golde et al., 2000). In contrast, Aβ peptides with the C-terminus ending at the amino acid 40 are more soluble and are reported to be more prevalent in vascular Aβ deposits (CAA) than in parenchymal Aβ plaques (Jarrett et al., 1993; Miller et al., 1993; Gravina et al., 1995; Iwatsubo et al., 1995; Akiyama et al., 1997; Harigaya et al., 2000; Guntert et al., 2006; Mann et al., 2018; Gkanatsiou et al., 2019). In addition, modified Aβ forms with *N*-terminus truncations are a significant proportion of total plaque-bound Aβ in AD and aged DS brains (Masters et al., 1985). *N*-terminus truncated Aβ can be modified further by the enzyme glutaminyl cyclase into forms with pyroglutamate at the 3rd amino acid (AβNpE3) or the 11th amino acid (AβNpE11; Cynis et al., 2008; Schilling et al., 2008; Morawski et al., 2104). Pyroglutamate-modified Aβ forms are believed to play a role in seeding or maturation of Aβ plaques; they are more resistant to proteolytic cleavage by peptidases, which may impede their clearance, and *in vitro* they accelerate fibril formation of unmodified forms (Saido et al., 1995; He and Barrow, 1999; Schilling et al., 2006; Gunn et al., 2010; Jawhar et al., 2011; Sullivan et al., 2011; Dammers et al., 2017; Michno et al., 2019). Both AβNpE3 and AβNpE11 forms contribute to Aβ plaques, however, AβNpE11 is restricted mainly to the innermost amyloid core (Sullivan et al., 2011) where it may be less accessible to peptidases as well as PET radioligands. The clinical significance of pyroglutamate Aβ is not known. Recent studies reported that high levels of insoluble Aβ42 forms, including AβNpE3-42, correlated with cognitive impairment across clinical stages of AD (Pivtoraiko et al., 2015; Abrahamson et al., 2016). Passive immunization with a pyroglutamate-3 Aβ IgG1 monoclonal antibody reduced amyloid plaque burden and improved behavior in APPswe/PS1$\rDelta$E9 mice (Frost et al., 2015). In a Phase 2 clinical trial of early AD, treatment with donanemab, a humanized IgG1 monoclonal antibody developed from the mouse monoclonal antibody mE8-IgG2a (Demattos et al., 2012) and specific for AβNpE3-42, reduced Aβ plaque burden and slowed cognitive decline (Mintun et al., 2021). Thus, AβNpE3-42 may be an important substrate for amyloid PET ligand retention, a biomarker for brain amyloidosis, and a therapeutic target.

In adults with DS, pyroglutamate-modified Aβ immunoreactivity was demonstrated in cortical Aβ plaques at the ages 30–40 years (but not younger), with greater abundance at ages 50–70 years (Lemere et al., 1996; Frost et al., 2013). Studies have also compared N-terminally truncated, pyroglutamate-modified Aβ forms to other forms of Aβ in DS brains using biochemical methods (Saido et al., 1995; Russo et al., 1997; Hosoda et al., 1998; Gkanatsiou et al., 2021) but none in relation to binding of PiB or related amyloid PET radioligands. In the current study, we quantified fibrillar forms of unmodified Aβ (Aβ42 and Aβ40) as well as pyroglutamate-modified Aβ (AβNpE3-42 and AβNpE3-40) and *in vitro* binding levels of ^3^H-PiB (as a proxy for PiB PET imaging) in postmortem homogenates of the frontal cortex and precuneus gray matter from a group of older adults with DS (age range: 43–63 years) compared to a group of sporadic AD cases (age range: 62–99 years) with a comparable degree of AD neuropathologic change.

## Materials and Methods

### Subjects

Frozen postmortem brain tissue specimens from the frontal cortex and the precuneus were obtained from 18 DS cases, provided by the University of California, Irvine Alzheimer’s Disease Research Center (UCI-ADRC) and Institute for Memory Impairments and Neurological Disorders, and from 17 sporadic AD cases in the University of Pittsburgh Alzheimer’s Disease Research Center (ADRC) brain bank. Clinical diagnosis of AD dementia utilized standard criteria (McKhann et al., 1984). Brain autopsy consent was obtained under a protocol approved by the Institutional Review Boards and the use of autopsy tissue for research was approved by the Committee for Oversight of Research and Clinical Training Involving Decedents (CORID) at the University of Pittsburgh and the University of California, Irvine. DS and AD brains were assessed for neocortical neuritic plaques and neurofibrillary pathology according to the National Institute on Aging-Alzheimer’s Association guidelines (Montine et al., 2012), using the Consortium to Establish a Registry for Alzheimer’s disease (CERAD) neuritic plaque scoring protocol (Mirra et al., 1991) and Braak staging for neurofibrillary pathology (Braak and Braak, 1991; Braak et al., 2006). The severity of CAA was evaluated separately in the frontal cortex and in the precuneus in both diagnostic groups, using Aβ immunohistochemistry with mouse monoclonal IgG clone NAB228 (37-4200, Thermo-Fisher, Waltham, MA) on 4% paraformaldehyde fixed tissue sections, on a four-point rating scale (0, none; 1, mild; 2, moderate; 3, severe) by two independent evaluators (EA and MI) adapted from published studies (Olichney et al., 1995; Arvanitakis et al., 2011). Demographic and neuropathological information of cases are detailed in Table 1. Frozen frontal cortex was not available for one AD case (AD-9). Frozen precuneus samples were not available for two DS cases (DS-10 and DS-11) and two AD cases (AD-8 and AD-10).

**Table 1:** Demographic and neuropathological characteristics of Down syndrome and Alzheimer’s disease cases.

Case code	Age (years)	Sex (M/F)	Neocortical neuritic plaques	Braak stage	CAA severity (frontal cortex)	CAA severity (precuneus cortex)
**Down syndrome**						
DS-1	49	M	Frequent	VI	None	None
DS-2	43	M	Frequent	VI	None	None
DS-3	57	F	Frequent	VI	None	Severe
DS-4	62	F	Frequent	VI	Mild	None
DS-5	45	F	Frequent	VI	Mild	Mild
DS-6	50	M	Frequent	VI	Mild	Mild
DS-7	57	F	Frequent	VI	Mild	Moderate
DS-8	55	F	Frequent	VI	Mild	Moderate
DS-9	52	F	Frequent	VI	Mild	Severe
DS-10	56	M	Frequent	VI	Moderate	Mild
DS-11	56	F	Frequent	VI	Moderate	Mild
DS-12	49	M	Frequent	VI	Moderate	Mild
DS-13	55	M	Frequent	VI	Moderate	Mild
DS-14	46	M	Frequent	VI	Moderate	Moderate
DS-15	50	F	Frequent	VI	Severe	Moderate
DS-16	63	F	Frequent	VI	Severe	Moderate
DS-17	58	M	Frequent	VI	Severe	Severe
DS-18	54	M	Frequent	VI	Severe	Severe
**Alzheimer’s disease**						
AD-1	85	M	Frequent	III/IV	None	None
AD-2	89	F	Frequent	VI	None	None
AD-3	91	M	Frequent	V	None	None
AD-4	77	M	Frequent	VI	None	Mild
AD-5	74	M	Frequent	VI	Mild	None
AD-6	67	F	Frequent	VI	Mild	None
AD-7	99	M	Frequent	V	Mild	None
AD-8	82	M	Frequent	VI	Mild	None
AD-9	91	F	Frequent	VI	Mild	None
AD-10	85	M	Frequent	VI	Mild	Mild
AD-11	62	M	Frequent	VI	Mild	Mid
AD-12	79	M	Frequent	VI	Mild	Mild
AD-13	84	M	Frequent	VI	Mild	Moderate
AD-14	77	M	Frequent	VI	Moderate	None
AD-15	88	M	Frequent	V	Moderate	Mild
AD-16	72	M	Frequent	VI	Severe	Moderate
AD-17	76	M	Frequent	V	Severe	Severe

### Tissue Preparation

Frozen gray matter samples were homogenized in 0.01 M sodium phosphate-buffered saline (PBS, pH 7.4) to a concentration of 300 mg wet brain tissue/mL. This homogenate is referred to as a “whole (unfractionated) tissue homogenate” and was used for the ^3^H-PiB binding assay. A protease inhibitor cocktail (AEBSF: 104 mM, aprotinin at 80 μM, bestatin at 4 mM, E-64 at 1.4 mM, leupeptin at 2 mM and pepstatin A at 1.5 mM; P8340, Sigma, St. Louis, MS; used at a 1:100 dilution) was added, and samples were then centrifuged at 100,000× *g* for 1 h at 4°C. The pellet was sonicated in 70% formic acid to solubilize Aβ fibrils. Samples were then centrifuged at 113,000× *g* for 1 h at 4°C. The supernatant containing the extracted, solubilized fibrillar Aβ fraction (hereafter referred to as “insoluble Aβ” and assayed by the ELISA) was then removed and neutralized to pH 7.4, divided into aliquots, and frozen at −80°C until testing was performed.

### Quantification of Aβ Peptide Levels

Solid-phase sandwich ELISA kits were used to measure AβNpE3-42 and AβNpE3-40 peptide levels (27716 and 27418, Immuno-Biological Laboratories, Minneapolis, MN). The AβNpE3-42 assay utilized a plate precoated with a capture antibody against the Aβ carboxy-terminal amino acid 42 (anti-human Aβ 38–42 rabbit polyclonal IgG). The AβNpE3-40 assay utilized a plate precoated with a capture antibody against the Aβ carboxy-terminal amino acid 40 (anti-human Aβ 35–40 mouse monoclonal IgG). A detection antibody for human AβNpE3 [anti-human AβN3pE (clone 8E1) mouse monoclonal IgG] was used in both assay kits. Solid phase sandwich ELISA kits were used to measure unmodified Aβ42 and Aβ40 peptide levels (KHB3441 and KHB3481, Thermo) on a plate precoated with a capture antibody directed against the unmodified amino terminus of Aβ, and detection antibodies specific for Aβ42 or Aβ40, respectively. Procedures were followed as outlined in the manufacturer’s instructions. Optical density values were read at 450 nm with a plate reader (SpectraMax i3x, Molecular Devices, San Jose, CA, USA) using SoftMax Pro software, Version 6.5.1 (Molecular Devices). Results were determined from standard curves that used synthetic human AβNpE3-40, AβNpE3-42, Aβ40, and Aβ42 and are expressed as picomoles per gram of wet tissue weight. Samples were run in duplicates, including both diagnostic groups and both brain regions in each experiment. Each sample was analyzed at least twice and the mean of the two assays was used to determine final values for each sample/analyte.

### ^3^H-PiB Binding Assay

Unfractionated whole brain tissue homogenates (described above) were diluted from 300 mg/ml to a concentration 10 mg/ml in PBS prior to the binding assay as previously described (Ikonomovic et al., 2008) with the exception of the fold-higher initial homogenate prepared in the current study. For determination of ^3^H-PiB binding, 1 nM ^3^H-PiB (American Radiolabeled Chemicals, St. Louis, MO, USA; specific activity 72.4 Ci/mmol) was incubated with 100 μg tissue in 1 ml PBS as described previously (Ikonomovic et al., 2008). Unlabeled PiB was dissolved in DMSO at 400 mM (to yield 51% DMSO) and this stock solution was diluted with PBS to achieve the desired concentration for the binding assay. Non-specific binding was defined as the number of counts remaining in the presence of 1 mM unlabeled PiB. The binding mixtures were filtered through a Whatman GF/B glass filter using a Brandel M24R cell harvester (Brandel, Gaithersburg, MD) and rapidly washed five times with 3 ml PBS. The filters were counted in Cytoscint-ES after thorough vortex mixing and resting overnight. Results were corrected for non-specific, non-displaceable binding in the presence of 1 mM PiB and expressed as picomoles ^3^H-PiB bound per gram of wet brain tissue weight in the homogenate.

### Statistical Analysis

Statistical analysis and graphs were performed using GraphPad PRISM Version 8 software (GraphPad, San Diego, CA, USA). The Kruskal-Wallis one-way analysis of variance was used to compare groups and pairwise comparisons were performed using Dunn’s multiple comparisons post test. The Spearman rank order correlation test was used to assess associations between two variables. Demographic and diagnostic neuropathological characteristics in the DS group were compared to the AD group using Student’s *t*-test and chi-square tests where appropriate. Significance was set at *P* < 0.05.

## Results

### Case Demographics and Neuropathological Characteristics

Individual case demographics and neuropathological characteristics are listed in Table 1. The DS group on average was younger than the AD group (DS: 53 ± 6 years; AD: 84 ± 10 years, *P* < 0.001; Table 1). Females were more represented in the DS group (nine females and nine males; 50%; *p* < 0.01) compared to the AD group (three females and 14 males; 18%).

The severity of AD neuropathological changes was similar between the two groups when compared by CERAD scores or by Braak staging, with all cases in the study having frequent neocortical neuritic plaques as well as neocortical stages of neurofibrillary pathology (Braak stages V or VI) with the exception of one AD case (AD-1) that was determined to be Braak stage III/IV (Table 1). The severity rating of CAA pathology was higher in the precuneus (*p* = 0.0156) and trended higher in the frontal cortex (*p* = 0.1990) in the DS group compared to the AD group (Table 1).

### Diagnostic Group and Brain Region Comparisons: ^3^H-PiB Binding, Unmodified Aβ42 and Aβ40, Pyroglutamate AβNpE3–42 and AβNpE3–40; Ratios of Aβ42/Aβ40 and AβNpE3–42/AβNpE3–40

**Figure 1:**
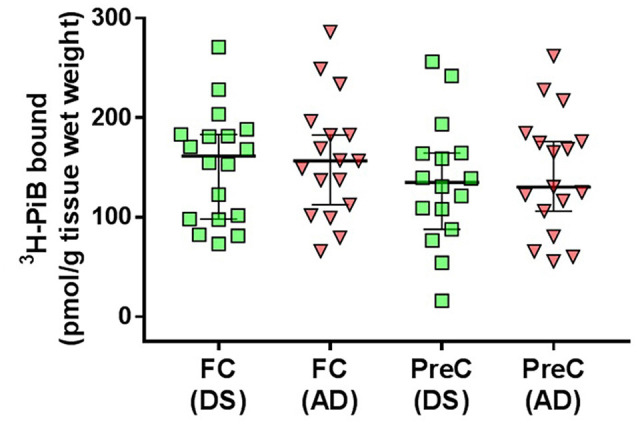
^3^H-PiB binding levels in tissue homogenates of the frontal cortex (FC) and precuneus (PreC) obtained postmortem from individuals with Down syndrome (DS) or Alzheimer’s disease (AD). Individual data points and group medians with 95% confidence intervals are illustrated in the graphs. Data were assessed using the Kruskal-Wallis one-way analysis of variance with Dunn’s multiple comparisons post test.

*in vitro*^3^H-PiB binding levels in the frontal cortex and in the precuneus in the DS group did not differ from ^3^H-PiB binding levels in the same regions, respectively, in the AD group (Figure 1, Table 2).

**Table 2:** Comparisons of ^3^H-PiB, unmodified Aβ, pryoglutamate modified Aβ, and ratios of Aβ forms across groups/regions and with each other.

Variable	Down syndrome (frontal cortex)	Alzheimer’s disease (frontal cortex)	Down syndrome (precuneus)	Alzheimer’s disease (precuneus)	Kruskal–Wallis statistic and *P* value	Pairwise comparisons (*P* < 0.05)
^3^H-PiB binding	152.2 ± 55.60 (161.4)	158.5 ± 59.94 (156.6)	135.1 ± 63.1 (134.9)	143.4 ± 60.86 (130.4)	1.35, *P* = 0.7128	n.s.
Aβ42	1,428 ± 373.2 (1,454)	1,274 ± 411.5 (1,252)	1,461 ± 534.3 (1,454)	1,119 ± 549.8 (919.5)	6.39, *P* = 0.0943	n.s.
Aβ40	2,868 ± 2,940 (1,337)	655.5 ± 893.0 (104.6)	5,149 ± 6,091 (2,261)	227.5 ± 404.5 (48.23)	28.46, *P* < 0.0001	FC-DS, PreC-DS > FC-AD, PreC-AD
AβNpE3-42	94.37 ± 14.97 (91.74)	90.5 ± 21.22 (90.49)	87.28 ± 16.22 (86.46)	81.25 ± 31.22 (83.25)	4.33, *P* = 0.2288	n.s.
AβNpE3-40	173 ± 208.3 (51.63)	33.26 ± 49.25 (8.38)	122.1 ± 147 (48.71)	33.1 ± 55.48 (5.88)	15.08, *P* = 0.0017	FC-DS, PreC-DS > FC-AD, PreC-AD
Kruskal	#x02013;Wallis statistic and *P* value	47.65, *P* < 0.0001	32.63, *P* < 0.0001	41.83, *P* < 0.0001	36.40, *P* < 0.0001		
Pairwise comparisons (*P* < 0.05)	Aβ42, Aβ40 > AβNpE3-42, AβNpE3-40	Aβ42 > Aβ40, AβNpE3-42, AβNpE3-40; Aβ40, AβNpE3-42 > AβNpE3-40	Aβ42, Aβ40 > AβNpE3-42, AβNpE3-40	Aβ42 > Aβ40, AβNpE3-40, AβNpE3-42; Aβ40, AβNpE3-42 > AβNpE3-40		
Aβ42/Aβ40	1.69 ± 1.82 (1.04)	13.29 ± 17.29 (4.0)	2.05 ± 3.05 (0.54)	43.44 ± 51.0 (13.95)	20.73, *P* = 0.0001	FC-DS, PreC-DS > FC-AD, PreC-AD
AβNpE3-42/AβNpE3-40	2.69 ± 3.05 (1.68)	18.98 ± 19.32 (12.61)	4.554 ± 6.939 (1.095)	29.37 ± 29.07 (10.12)	13.36, *P* = 0.0035	FC-DS, PreC-DS > FC-AD, PreC-AD
Aβ42/AβNpE3-42	16.01 ± 2.12 (15.86)	13.92 ± 4.40 (13.51)	15.83 ± 4.22 (15.11)	14.50 ± 8.37 (11.30)	10.0, *P* = 0.0185	FC-DS > FC-AD, PreC-AD PreC-DS > FC-AD
Aβ40/AβNpE3-40	25.73 ± 30.43 (18.72)	15.69 ± 10.38 (13.02)	71.43 ± 153.7 (33.16)	10.19 ± 11.96 (8.48)	28.46, *P* < 0.0001	FC-DS > PreC-AD; PreC-DS > FC-AD, PreC-AD

*Horizontal comparisons: AβNpE3-40, AβNpE3-42, and the AβNpE3-42/AβNpE3-40 ratio, Aβ40, Aβ42, and the Aβ42/Aβ40 ratio, and ^3^H-PiB binding levels in Down syndrome compared to Alzheimer’s disease in the frontal cortex and in the precuneus. Vertical comparisons: AβNpE3-40, AβNpE3-42, Aβ40, and Aβ42 levels separately in the frontal cortex (FC) and precuneus cortex (PreC) in Down syndrome and in Alzheimer’s disease. Units are pmol/g tissue wet weight and arithmetic means ± standard deviations and medians are shown. Comparisons were made using the Kruskal-Wallis one-way analysis of variance with Dunn’s post test for multiple comparisons*.

**Figure 2:**
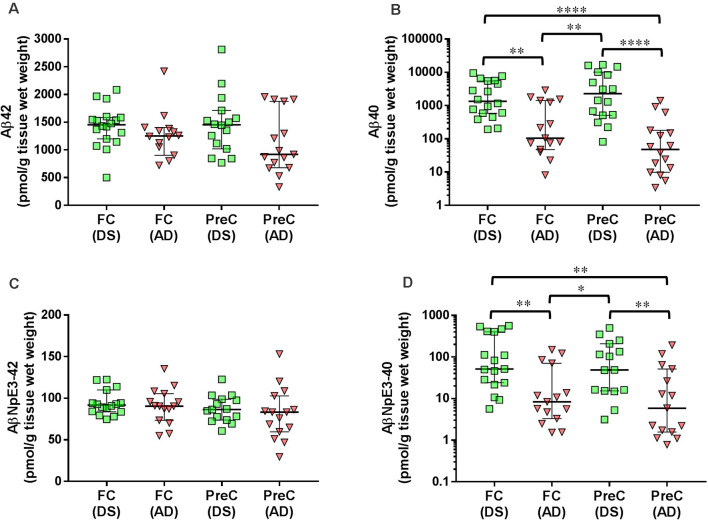
The concentration of Aβ42 **(A)**, Aβ40 **(B)**, AβNpE3-42 **(C)**, and AβNpE3-40 **(D)** in tissue homogenates of the frontal cortex (FC) and precuneus (PreC) obtained postmortem from individuals with Down syndrome (DS) or Alzheimer’s disease (AD). Individual data points and group medians with 95% confidence intervals are illustrated in the graphs. The y-axis in panels B and D have been log transformed to better illustrate the spread of individual data points. Data were assessed using the Kruskal-Wallis one-way analysis of variance with Dunn’s multiple comparisons post test. Significant differences between the two groups that were identified by the post test are indicated by brackets and asterisks. *P < 0.05; **P < 0.01; ****P < 0.0001.

**Figure 3:**
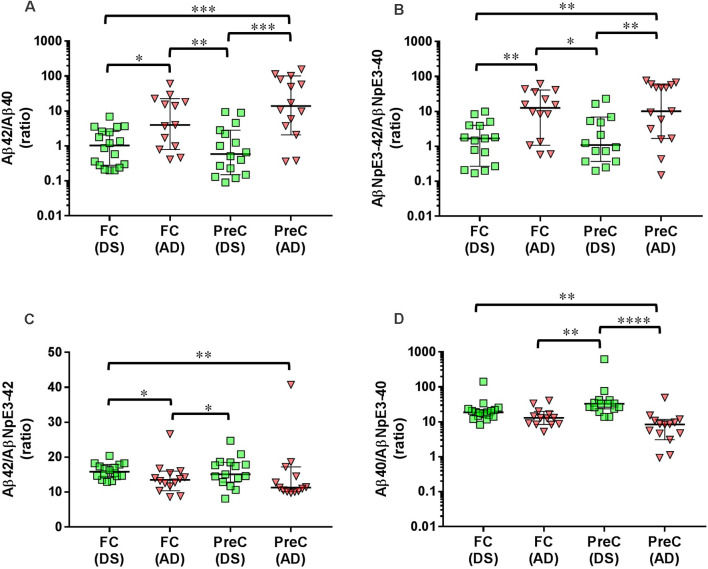
Ratios of unmodified Aβ42/Aβ40 **(A)** and pyroglutamate AβNpE3-42/NpE3–40 **(B)**, and ratios of unmodified and pyroglutamate forms of Aβ ending at amino acid 42 **(C)** or at amino acid 40 **(D)** in tissue homogenates of the frontal cortex (FC) and precuneus (PreC) obtained postmortem from individuals with Down syndrome (DS) and Alzheimer’s disease (AD). Individual data points and group medians with 95% confidence intervals are illustrated in the graphs. The y-axis in panels A, B, and D have been log transformed to better illustrate the spread of individual data points. The data were assessed using the Kruskal-Wallis one-way analysis of variance with Dunn’s multiple comparisons post test. Significant differences between the two groups that were identified by the post test are indicated by brackets and asterisks. *P < 0.05; **P < 0.01; ***P < 0.01; ****P < 0.0001.

In both the frontal cortex and the precuneus, levels of unmodified Aβ42 and pyroglutamate AβNpE3-42 were not statistically different between DS and AD groups (Figures 2A–C, Table 2). Unmodified Aβ40 and pyroglutamate AβNpE3-40 levels in the frontal cortex and in the precuneus were significantly higher in the DS group compared to the AD group (Figures 2B,D, Table 2). The ratios of unmodified Aβ42/Aβ40 levels and pyroglutamate AβNpE3-42/AβNpE3-40 levels in the frontal cortex and in the precuneus were significantly lower in the DS group compared to the AD group (Figures 3A,B, Table 2).

### Comparisons of Unmodified and Pyroglutamate-Modified Aβ Forms Within Each Brain Region in Each Diagnostic Group

In both the frontal cortex and the precuneus from the DS group, unmodified Aβ42 and Aβ40 were at similar levels and both were higher than AβNpE3-42 and AβNpE3-40 levels, which were also at similar levels in this group (Table 2). In the frontal cortex and in the precuneus from the AD group, unmodified Aβ42 levels were higher than Aβ40, AβNpE3-40, and AβNpE3-42 levels, and both Aβ40 and AβNpE3-42 levels were higher than AβNpE3-40 levels (Table 2).

### Ratios of Aβ42/AβNpE3–42 and Aβ40/AβNpE3–40 in the DS Group Compared to the AD Group

In the DS group, the ratio of Aβ42/AβNpE3-42 in the frontal cortex was higher, while in the precuneus the ratio trended higher, compared to the AD group (Figure 3C, Table 2). The ratio of Aβ40/AβNpE3-40 levels in the precuneus was higher in the DS group compared to the AD group, while in the frontal cortex the ratio trended higher in the DS group (Figure 3D, Table 2).

### Associations of Unmodified Aβ Levels With Pyroglutamate-Modified Aβ Levels, Both Aβ Forms With ^3^H-PiB Binding Levels, and Both Aβ Forms and ^3^H-PiB Binding Levels With CAA Severity in Down Syndrome and Alzheimer’s Disease Groups

For correlation analyses within each diagnostic group, data from the frontal cortex and the precuneus were combined. We observed significant associations between levels of Aβ42 and AβNpE3-42 and between levels of Aβ40 and AβNpE3-40 in each diagnostic group (Table 3).

**Table 3:** Associations of unmodified Aβ levels with pyroglutamate-modified Aβ levels, both Aβ forms with ^3^H-PiB binding levels, and both Aβ forms with CAA severity in Down syndrome and Alzheimer’s disease groups.

Comparison/Group	Down syndrome	Alzheimer’s disease
Unmodified Aβ to pyroglutamate-modified Aβ (same *C*-terminus)	*Spearman r (P* value)	*Spearman r (P* value)
Aβ42 and AβNpE3-42	*0.5476 (0.0014)*	*0.6408 (<0.0001)*
Aβ40 and AβNpE3-40	*0.8292 (<0.0001)*	*0.8626 (<0.0001)*
Aβ forms to ^3^H-PiB	*Spearman r (P* value)	*Spearman r (P* value)
Aβ42 and ^3^H-PiB	*0.4197 (0.0135)*	*0.2852 (0.1337)*
Aβ40 and ^3^H-PiB	0.2825 (0.1055)	−0.0097 (0.9588)
AβNpE3-42 and ^3^H-PiB	*0.3661 (0.0428)*	*0.4459 (0.0135)*
AβNpE3-40 and ^3^H-PiB	*0.4377 (0.0122)*	−0.0269 (0.8877)
Aβ42/Aβ40 ratio and ^3^H-PiB	−0.2610 (0.1360)	0.2717 (0.1704)
AβNpE3-42/AβNpE3-40 and ^3^H-PiB	*−0.5097 (0.0047)*	0.0631 (0.7452)
Aβ forms and ^3^H-PiB to CAA severity	*Spearman r (P* value)	*Spearman r (P* value)
Aβ42 and CAA severity	*0.4088 (0.0164)*	−0.2090 (0.2766)
Aβ40 and CAA severity	0.7786 (<0.0001)	*0.5397 (0.0017)*
AβNpE3-42 and CAA severity	*−0.0092 (0.9608)*	*−0.4759 (0.0079)*
AβNpE3-40 and CAA severity	*0.7672 (<0.0001)*	*0.3273 (0.0474)*
Aβ42/Aβ40 and CAA severity	*−0.7739 (<0.0001)*	*−0.5134 (0.0062)*
AβNpE3-42/AβNpE3-40 and CAA severity	*−0.8394 (<0.0001)*	*−0.4108 (0.0269)*
^3^H-PiB and CAA severity	*0.3391 (0.0498)*	−0.0419 (0.8141)

*For each correlation analysis, data from the frontal cortex and the precuneus were combined. The Spearman rank order correlation test was used to examine associations among variables. Associations meeting criteria for significance (*P* < 0.05) are italicized*.

There was a significant association of unmodified Aβ42 levels with ^3^H-PiB levels in the DS group and a similar trend was present in the AD group (Table 3). No associations were observed between unmodified Aβ40 levels and ^3^H-PiB levels in either diagnostic group. We observed significant associations of AβNpE3-42 levels with ^3^H-PiB levels in both diagnostic groups and a significant association of AβNpE3-40 levels with ^3^H-PiB levels in the DS group but not in the AD group (Table 3). No associations were observed between the ratio of unmodified Aβ forms and ^3^H-PiB levels in either diagnostic group. There was a significant association of the ratio AβNpE3-42/AβNpE3-40 with ^3^H-PiB levels in the DS group but not in the AD group (Table 3).

In both diagnostic groups, there were significant associations of Aβ40 and AβNpE3-40 levels with CAA severity and of ratios Aβ42/Aβ40 and AβNpE3-42/AβNpE3-40 with CAA severity (Table 3). Greater CAA severity correlated with higher ^3^H-PiB binding levels in the DS group, but not in the AD group (Table 3).

## Discussion

The extent of interaction between PiB, or related amyloid-binding radiopharmaceuticals for PET imaging, with different unmodified Aβ forms or post-translationally truncated and pyroglutamate-modified Aβ forms in pathological amyloid deposits in cortical regions from DS and AD brains is not well understood. Better characterization of these interactions could facilitate the interpretation of amyloid PET imaging studies and identify targets for therapy. DS patients develop amyloid pathology and are likely to show positive amyloid PET scans, by age 40. Since the pyroglutamate modification is believed to drive Aβ fibrillization and deposition in amyloid plaques (Jawhar et al., 2011), in the present study we undertook a quantitative ELISA analysis of insoluble (fibrillar) pools of unmodified Aβ42 and Aβ40 forms as well as *N*-terminus truncated and pyroglutamate-modified AβNpE3-42 and AβNpE3-40 forms in the frontal cortex and the precuneus from adult DS cases compared to a group of sporadic AD cases with similar levels of neocortical neuritic plaques and neurofibrillary tangle pathology. Additionally, we assayed ^3^H-PiB binding levels in the same homogenates used for the ELISA studies. We found that in both the frontal cortex and the precuneus regions, Aβ42, AβNpE3-42, and ^3^H-PiB binding levels did not differ significantly between the two diagnostic groups. In contrast, DS cases had significantly higher Aβ40 and AβNpE3-40 levels, and lower Aβ42/Aβ40 and AβNpE3-42/AβNpE3-40 ratios in these cortical regions, compared to AD cases.

Genetic predisposition for premature pathological aging with early-onset of Aβ plaque accumulation in DS relative to sporadic AD (Teller et al., 1996; Mori et al., 2002; Zigman et al., 2002, 2008) could result in a greater abundance of fibrillar Aβ deposits in brains of adults with DS when compared to individuals in the early stages of AD. In agreement with this, we previously reported higher levels of insoluble unmodified Aβ42, and a greater burden of mature amyloid plaques, in the frontal cortex from adult individuals with DS (age range: 43–63 years) compared to cases with mild-moderate AD from the Rush Religious Order Study (age range: 77–101 years; Abrahamson et al., 2019). In contrast, our current study demonstrated that in cases from the same DS cohort, neocortical levels of insoluble unmodified Aβ42 were not different from a group of late-stage AD cases in our ADRC autopsy cohort (age range: 62–99 years). Cortical levels of insoluble pyroglutamate AβNpE3-42 were also similar between the DS group and the late stage AD group in the current study. The propensity for the Aβ forms ending at amino acid 42 to aggregate into amyloid fibrils and deposit early in the process of amyloid plaque formation could explain these observations. Specifically, our DS subjects were above the age of 40 years and exhibited a high level of amyloid plaque pathology by CERAD scores for neocortical neuritic plaques that were similar to late-stage AD (Wisniewski et al., 1985; Mann and Esiri, 1989), thus it is possible that levels of fibrillar Aβx-42 forms reach a plateau early in the pathological progression of DS and AD. In contrast, Aβx-40 forms clearly distinguished DS and AD groups in our current study; in both cortical regions examined, we observed significantly higher levels of Aβ40 and AβNpE3-40 in the DS group compared to the late-stage AD group and, as a result, DS cases had lower ratios of Aβ42/40 and AβNpE3-42/AβNpE3-40 when compared to late-stage AD cases. Higher levels of Aβ40 and AβNpE3-40 in the frontal cortex of DS cases compared to AD cases were also reported in a study of five DS individuals (age range: 53–67) and 14 AD cases (age 66–86; Hosoda et al., 1998). Another major finding of our current study is that compared to AD, the DS group had lower proportions of pyroglutamate relative to unmodified Aβ forms. This could be explained by a shorter residence time of Aβ deposits in DS brains when compared to brains of older sporadic AD cases with end-stage pathology. Different proportions of pyroglutamate-modified Aβ and unmodified Aβ in DS compared to preclinical (pathological aging) and clinical AD might affect their detection by amyloid PET. This could have influenced the findings of a longitudinal ^11^C-PiB PET study which reported slower progression of the frontal cortex and precuneus amyloid pathology in nondemented young adults with DS (mean age 37 years) when compared to nondemented elderly (mean age 73 years; Tudorascu et al., 2019). In contrast to our findings, Hosoda and colleagues reported higher levels of AβNpE3-42 compared to unmodified Aβ1–42 in their DS group (Hosoda et al., 1998). The small number of DS cases and large individual variability of pyroglutamate-modified Aβ levels in the latter report make it difficult to explain this discrepancy.

The preponderance of Aβ40 levels in unmodified and pyroglutamate-modified forms in our DS group appears to be influenced by greater severity of CAA, despite comparable levels of mature amyloid plaques (frequent neocortical neuritic plaques) in the DS and AD groups. This is supported by our observations that levels of Aβ40 and AβNpE3-40 forms (and ratios of Aβ42/Aβ40 and AβNpE3-42/AβNpE3-40) were associated strongly with CAA severity in the DS group, while in the AD group these associations were much weaker. Previous studies reported that CAA is a significant contributor to the neuropathology of DS and is observed more frequently in DS adults over 45–50 years of age than in people with sporadic AD and normal elderly controls (Vinters, 1987; Wilcock et al., 2016; Carmona-Iragui et al., 2017; Head et al., 2017; Davidson et al., 2018). Our results are also consistent with studies reporting that Aβ40 is the primary constituent of vascular amyloid in AD and DS (Miller et al., 1993; Iwatsubo et al., 1995; Harigaya et al., 2000; Guntert et al., 2006; Mann et al., 2018; Gkanatsiou et al., 2019).

We observed that DS and late-stage AD groups had similar levels of ^3^H-PiB binding in the frontal cortex and in the precuneus. Although DS cases had higher levels of Aβ forms ending at carboxy terminus amino acid 40 and greater severity of CAA, which correlated strongly with greater ^3^H-PiB binding, the lack of differences in ^3^H-PiB binding between DS and AD groups appears to be influenced more by these two groups having similar levels of unmodified and pyroglutamate-modified Aβ42 forms. This is in agreement with observations from PiB PET imaging-autopsy studies of AD and *in vitro* analyses of synthetic Aβ that PiB binding is influenced primarily by the Aβ42 form (Ikonomovic et al., 2008, 2020; Yamin and Teplow, 2017) which was reported as the initial and dominant Aβ form in amyloid plaques in AD and DS (Miller et al., 1993; Iwatsubo et al., 1994, 1995, 1996; Lemere et al., 1996). Interestingly, the strongest correlate of (higher) ^3^H-PiB binding was the (lower) ratio of insoluble AβNpE3-42/AβNpE3-40 in DS, but not in the AD group. However, higher levels of insoluble AβNpE3-40 and Aβ40, as well as greater severity of CAA, do not appear to be the main determinants of ^3^H-PiB binding levels in DS cases when the overall parenchymal plaque pathology burden is high.

Several potential limitations should be considered in the current study. The commercial ELISA kits we used for the detection of unmodified Aβ forms have been widely applied and reported in published studies. The specificity of their *N*-terminus (detection) antibody is defined in the range of Aβ amino acids 1–16, and this overlaps the range recognized by the well-characterized monoclonal IgG clone 6E10 (Kim et al., 1990). Thus, we cannot be confident that these kits measure exclusively the intact “full-length” Aβ1–40 and Aβ1–42 forms, because theoretically, they could detect some Aβ forms truncated at the proximal portion of the *N*-terminus. However, *N*-terminus truncated and pyroglutamate-modified forms of Aβ likely undergo additional molecular and conformational modifications and this could interfere with the binding of *N*-terminus-directed antibodies to epitopes that overlap, or are near to, the pyroglutamate modification. Secondly, consistent with brain tissue sampling in a previous analysis of unmodified and pyroglutamate-modified Aβ concentrations in DS and AD (Hosoda et al., 1998), cortical samples in our study were stripped of the leptomeningeal vessels but they included intraparenchymal vasculature, so the ELISA and ^3^H-PiB binding assays measured insoluble Aβ from the combined pool of amyloid plaques as well as capillary and arterial CAA. This approach is consistent with ^11^C-PiB PET and related amyloid PET radioligands lacking selectivity to distinguish Aβ plaques from CAA (Bacskai et al., 2007; Johnson et al., 2007; Lockhart et al., 2007; Dierksen et al., 2010; Ly et al., 2010; Sabbagh et al., 2011; Ducharme et al., 2013; Murray et al., 2015; Seo et al., 2017; Charidimou et al., 2018; Planton et al., 2020). Further studies will require biochemical analyses of unmodified and pyroglutamate-modified Aβ forms and ^3^H-PiB binding in isolated microvessels compared to vessel-free brain parenchyma extracts from DS brains as has been done previously in samples from AD brains (Roher et al., 1993; Kuo et al., 1997; Bourassa et al., 2019) as well as isolated plaque cores in both diagnostic groups to extend previous studies (Allsop et al., 1986; Roher et al., 1993). Lastly, as in most brain banks, the frozen tissue samples for biochemical assays and fixed tissue samples for neuropathological workup were not from the same hemisphere. We assumed, in this study, that neuropathological findings from one hemisphere informed us about the overall brain pathology including the opposite hemisphere from which our samples for ELISA and ^3^H-PiB binding assays were obtained.

In summary, our study demonstrates that compared to late-stage AD cases, older adults with DS have similar levels of ^3^H-PiB binding in the frontal cortex and in the precuneus. This is consistent with the observation that both groups had frequent neocortical neuritic plaques and similar levels of Aβ42 and AβNpE3-42 forms in these brain regions. The DS group had more severe CAA pathology and significantly higher levels of Aβ40 and AβNpE3-40 forms in the same cortical regions, however, this was not the key determinant of PiB binding. The presence of CAA is significant because it can impact clinical presentation. For example, CAA independently affects cognition, and when present in conjunction with AD pathology it can result in more severe cognitive impairment (Pfeifer et al., 2002). Interestingly, while the cerebrovascular disease is considered a “second hit” that contributes to the clinical manifestation of AD (Provenzano et al., 2013), DS individuals may have a predisposition that protects against cardiovascular risk factors (Murdoch et al., 1977; Pucci et al., 2016; Lott and Head, 2019), but this protection may be offset by the development of severe CAA pathology in the DS brain. PiB binds to fibrillar Aβ in both parenchymal and vascular (CAA) deposits, and these two pathologies cannot be distinguished unequivocally on amyloid PET using PiB or related amyloid-binding radioligands (Bacskai et al., 2007; Lockhart et al., 2007; Dierksen et al., 2010; Sabbagh et al., 2011; Ducharme et al., 2013). Instead, the presence of CAA in the clinical setting is suspected only if there is a predominance of occipital signal on amyloid PET scans (Johnson et al., 2007; Greenberg et al., 2008; Seo et al., 2017) and higher frequencies of microbleeds, hemorrhagic lesions, or ischemic lesions detected by MR imaging (Dierksen et al., 2010; Ly et al., 2010; Viswanathan and Greenberg, 2011; Yamada, 2015). PET radiotracers selective for CAA are still under development (Abrahamson et al., 2021) and will be critical to incorporate into the neuroimaging biomarker panel for DS, to monitor Aβ deposition in the cerebral vasculature relative to PET measures of total (parenchymal and vascular) amyloid.

## Data Availability Statement

The datasets generated in the current study are available from the corresponding author on reasonable request.

## Ethics Statement

The studies involving human participants were reviewed and approved by The Institutional Review Board and the Committee for Oversight of Research and Clinical Training Involving Decedents, University of Pittsburgh and University of California, Irvine. Written informed consent for participation was not required for this study in accordance with the national legislation and the institutional requirements.

## Author Contributions

EA, VV, BH, IL, EH, and MI contributed to the design and implementation of the research. VP, TR, EA, VV, and MI contributed to the analysis of the results. All authors contributed to the writing of the manuscript.

## Conflict of Interest

The authors declare that the research was conducted in the absence of any commercial or financial relationships that could be construed as a potential conflict of interest.

## Publisher’s Note

All claims expressed in this article are solely those of the authors and do not necessarily represent those of their affiliated organizations, or those of the publisher, the editors and the reviewers. Any product that may be evaluated in this article, or claim that may be made by its manufacturer, is not guaranteed or endorsed by the publisher.

## References

[B1] Abrahamson E. E. Head E. Lott I. T. Handen B. L. Mufson E. J. Christian B. T.. (2019). Neuropathological correlates of amyloid PET imaging in Down syndrome. Dev. Neurobiol. 79, 750–766. 10.1002/dneu.22713 31379087 PMC6892598

[B2] Abrahamson E. E. Kruszka G. I. Mi Z. R. P. W. Debnath M. L. Klunk W. E. Mufson E. J.. (2016). Pyroglutamate and full-length amyloid-B concentrations in the superior frontal cortex across clinical stages of Alzheimer’s disease. Alzheimers Dement. 12, P742–P743. 10.1016/j.jalz.2016.06.1548

[B3] Abrahamson E. E. Stehouwer J. S. Vazquez A. L. Huang G. F. Mason N. S. Lopresti B. J.. (2021). Development of a PET radioligand selective for cerebral amyloid angiopathy. Nucl. Med. Biol. 92, 85–96. 10.1016/j.nucmedbio.2020.05.001 32471773 PMC8788879

[B4] Akiyama H. Mori H. Sahara N. Kondo H. Ikeda K. Nishimura T.. (1997). Variable deposition of amyloid β-protein (A β) with the carboxy-terminus that ends at residue valine40 (A β 40) in the cerebral cortex of patients with Alzheimer’s disease: a double-labeling immunohistochemical study with antibodies specific for a β 40 and the A β that ends at residues alanine42/threonine43 (A β 42). Neurochem. Res. 22, 1499–1506. 10.1023/a:1021910729963 9357016

[B5] Allsop D. Kidd M. Landon M. Tomlinson A. (1986). Isolated senile plaque cores in Alzheimer’s disease and Down’s syndrome show differences in morphology. J. Neurol. Neurosurg Psychiatry 49, 886–892. 10.1136/jnnp.49.8.886 2943873 PMC1028949

[B6] Annus T. Wilson L. R. Acosta-Cabronero J. Cardenas-Blanco A. Hong Y. T. Fryer T. D.. (2017). The Down syndrome brain in the presence and absence of fibrillar β-amyloidosis. Neurobiol. Aging 53, 11–19. 10.1016/j.neurobiolaging.2017.01.009 28192686 PMC5391869

[B7] Annus T. Wilson L. R. Hong Y. T. Acosta-Cabronero J. Fryer T. D. Cardenas-Blanco A.. (2016). The pattern of amyloid accumulation in the brains of adults with down syndrome. Alzheimers Dement. 12, 538–545. 10.1016/j.jalz.2015.07.490 26362596 PMC4867786

[B8] Arvanitakis Z. Leurgans S. E. Wang Z. Wilson R. S. Bennett D. A. Schneider J. A.. (2011). Cerebral amyloid angiopathy pathology and cognitive domains in older persons. Ann. Neurol. 69, 320–327. 10.1002/ana.22112 21387377 PMC3228518

[B9] Bacskai B. J. Frosch M. P. Freeman S. H. Raymond S. B. Augustinack J. C. Johnson K. A.. (2007). Molecular imaging with pittsburgh compound b confirmed at autopsy: a case report. Arch. Neurol. 64, 431–434. 10.1001/archneur.64.3.431 17353389

[B10] Bourassa P. Tremblay C. Schneider J. A. Bennett D. A. Calon F. (2019). β-amyloid pathology in human brain microvessel extracts from the parietal cortex: relation with cerebral amyloid angiopathy and Alzheimer’s disease. Acta Neuropathol. 137, 801–823. 10.1007/s00401-019-01967-4 30729296 PMC6483878

[B11] Braak H. Alafuzoff I. Arzberger T. Kretzschmar H. Del Tredici K. (2006). Staging of Alzheimer disease-associated neurofibrillary pathology using paraffin sections and immunocytochemistry. Acta Neuropathol. 112, 389–404. 10.1007/s00401-006-0127-z 16906426 PMC3906709

[B12] Braak H. Braak E. (1991). Neuropathological stageing of Alzheimer-related changes. Acta Neuropathol. 82, 239–259. 10.1007/BF00308809 1759558

[B13] Buckner R. L. Snyder A. Z. Shannon B. J. LaRossa G. Sachs R. Fotenos A. F.. (2005). Molecular, structural and functional characterization of Alzheimer’s disease: evidence for a relationship between default activity, amyloid and memory. J. Neurosci. 25, 7709–7717. 10.1523/JNEUROSCI.2177-05.2005 16120771 PMC6725245

[B14] Carmona-Iragui M. Balasa M. Benejam B. Alcolea D. Fernandez S. Videla L.. (2017). Cerebral amyloid angiopathy in down syndrome and sporadic and autosomal-dominant Alzheimer’s disease. Alzheimers Dement. 13, 1251–1260. 10.1016/j.jalz.2017.03.007 28463681 PMC5660938

[B15] Charidimou A. Farid K. Tsai H. H. Tsai L. K. Yen R. F. Baron J. C.. (2018). Amyloid-PET burden and regional distribution in cerebral amyloid angiopathy: a systematic review and meta-analysis of biomarker performance. J. Neurol. Neurosurg. Psychiatry 89, 410–417. 10.1136/jnnp-2017-316851 29070646

[B16] Chen G. F. Xu T. H. Yan Y. Zhou Y. R. Jiang Y. Melcher K.. (2017). Amyloid β: structure, biology and structure-based therapeutic development. Acta Pharmacol. Sin. 38, 1205–1235. 10.1038/aps.2017.28 28713158 PMC5589967

[B17] Cody K. A. Piro-Gambetti B. Zammit M. D. Christian B. T. Handen B. L. Klunk W. E.. (2020). Association of sleep with cognition and β amyloid accumulation in adults with down syndrome. Neurobiol. Aging 93, 44–51. 10.1016/j.neurobiolaging.2020.04.018 32447011 PMC7380565

[B18] Cohen A. D. McDade E. Christian B. Price J. Mathis C. Klunk W.. (2018). Early striatal amyloid deposition distinguishes Down syndrome and autosomal dominant Alzheimer’s disease from late-onset amyloid deposition. Alzheimers Dement. 14, 743–750. 10.1016/j.jalz.2018.01.002 29477284 PMC5994364

[B19] Cohen A. D. Rabinovici G. D. Mathis C. A. Jagust W. J. Klunk W. E. Ikonomovic M. D. (2012). Using Pittsburgh Compound B for *in vivo* PET imaging of fibrillar amyloid-β. Adv. Pharmacol. 64, 27–81. 10.1016/B978-0-12-394816-8.00002-7 22840744 PMC3542972

[B20] Cole J. H. Annus T. Wilson L. R. Remtulla R. Hong Y. T. Fryer T. D.. (2017). Brain-predicted age in Down syndrome is associated with β amyloid deposition and cognitive decline. Neurobiol. Aging 56, 41–49. 10.1016/j.neurobiolaging.2017.04.006 28482213 PMC5476346

[B21] Creekmore B. C. Chang Y. W. Lee E. B. (2021). The cryo-EM effect: structural biology of neurodegenerative disease aggregates. J. Neuropathol. Exp. Neurol. 80, 514–529. 10.1093/jnen/nlab039 33970243 PMC8177849

[B22] Cynis H. Scheel E. Saido T. C. Schilling S. Demuth H. U. (2008). Amyloidogenic processing of amyloid precursor protein: evidence of a pivotal role of glutaminyl cyclase in generation of pyroglutamate-modified amyloid-β. Biochemistry 47, 7405–7413. 10.1021/bi800250p 18570439

[B23] Dammers C. Schwarten M. Buell A. K. Willbold D. (2017). Pyroglutamate-modified Aβ (3–42) affects aggregation kinetics of Aβ (1–42) by accelerating primary and secondary pathways. Chem. Sci. 8, 4996–5004. 10.1039/c6sc04797a 28970886 PMC5612032

[B24] Davidson Y. S. Robinson A. Prasher V. P. Mann D. M. A. (2018). The age of onset and evolution of braak tangle stage and thal amyloid pathology of Alzheimer’s disease in individuals with down syndrome. Acta Neuropathol. Commun. 6:56. 10.1186/s40478-018-0559-4 29973279 PMC6030772

[B25] Demattos R. B. Lu J. Tang Y. Racke M. M. Delong C. A. Tzaferis J. A.. (2012). A plaque-specific antibody clears existing β-amyloid plaques in Alzheimer’s disease mice. Neuron 76, 908–920. 10.1016/j.neuron.2012.10.029 23217740

[B26] Dickson D. W. (2005). Required techniques and useful molecular markers in the neuropathologic diagnosis of neurodegenerative diseases. Acta Neuropathol. 109, 14–24. 10.1007/s00401-004-0950-z 15645265

[B27] Dickson D. W. (1997). The pathogenesis of senile plaques. J. Neuropathol. Exp. Neurol. 56, 321–339. 10.1097/00005072-199704000-00001 9100663

[B28] Dierksen G. A. Skehan M. E. Khan M. A. Jeng J. Nandigam R. N. Becker J. A.. (2010). Spatial relation between microbleeds and amyloid deposits in amyloid angiopathy. Ann. Neurol. 68, 545–548. 10.1002/ana.22099 20865701 PMC2964411

[B29] Ducharme S. Guiot M. C. Nikelski J. Chertkow H. (2013). Does a positive pittsburgh compound B scan in a patient with dementia equal Alzheimer disease? JAMA Neurol. 70, 912–914. 10.1001/jamaneurol.2013.420 23689280

[B30] Frost J. L. Le K. X. Cynis H. Ekpo E. Kleinschmidt M. Palmour R. M.. (2013). Pyroglutamate-3 amyloid-β deposition in the brains of humans, non-human primates, canines and Alzheimer disease-like transgenic mouse models. Am. J. Pathol. 183, 369–381. 10.1016/j.ajpath.2013.05.005 23747948 PMC3730768

[B31] Frost J. L. Liu B. Rahfeld J. U. Kleinschmidt M. O’Nuallain B. Le K. X.. (2015). An anti-pyroglutamate-3 Aβ vaccine reduces plaques and improves cognition in APPswe/PS1DeltaE9 mice. Neurobiol. Aging 36, 3187–3199. 10.1016/j.neurobiolaging.2015.08.021 26453001 PMC4641825

[B32] Gkanatsiou E. Portelius E. Toomey C. E. Blennow K. Zetterberg H. Lashley T.. (2019). A distinct brain β amyloid signature in cerebral amyloid angiopathy compared to Alzheimer’s disease. Neurosci. Lett. 701, 125–131. 10.1016/j.neulet.2019.02.033 30807796

[B33] Gkanatsiou E. Sahlin C. Portelius E. Johannesson M. Soderberg L. Falting J.. (2021). Characterization of monomeric and soluble aggregated Aβ in Down’s syndrome and Alzheimer’s disease brains. Neurosci. Lett. 754:135894. 10.1016/j.neulet.2021.135894 33848613

[B34] Golde T. E. Eckman C. B. Younkin S. G. (2000). Biochemical detection of Aβ isoforms: implications for pathogenesis, diagnosis and treatment of Alzheimer’s disease. Biochim. Biophys. Acta 1502, 172–187. 10.1016/s0925-4439(00)00043-0 10899442

[B35] Gravina S. A. Ho L. Eckman C. B. Long K. E. Otvos L. Jr. (1995). Amyloid β protein (A β) in Alzheimer’s disease brain. Biochemical and immunocytochemical analysis with antibodies specific for forms ending at a β 40 or A β 42(43). J. Biol. Chem. 270, 7013–7016. 10.1186/s40478-021-01225-3 7706234

[B36] Greenberg S. M. Grabowski T. Gurol M. E. Skehan M. E. Nandigam R. N. Becker J. A.. (2008). Detection of isolated cerebrovascular β-amyloid with Pittsburgh compound B. Ann. Neurol. 64, 587–591. 10.1002/ana.21528 19067370 PMC2605158

[B37] Gunn A. P. Masters C. L. Cherny R. A. (2010). Pyroglutamate-Aβ: role in the natural history of Alzheimer’s disease. Int. J. Biochem. Cell Biol. 42, 1915–1918. 10.1016/j.biocel.2010.08.015 20833262

[B38] Guntert A. Dobeli H. Bohrmann B. (2006). High sensitivity analysis of amyloid-β peptide composition in amyloid deposits from human and PS2APP mouse brain. Neuroscience 143, 461–475. 10.1016/j.neuroscience.2006.08.027 17008022

[B39] Handen B. L. Cohen A. D. Channamalappa U. Bulova P. Cannon S. A. Cohen W. I.. (2012). Imaging brain amyloid in nondemented young adults with Down syndrome using pittsburgh compound B. Alzheimers Dement. 8, 496–501. 10.1016/j.jalz.2011.09.229 23102120 PMC3532743

[B40] Handen B. L. Lott I. T. Christian B. T. Schupf N. S. O. B. Mapstone M. Fagan A. M.. (2020). The Alzheimer ’s biomarker consortium-down syndrome: rationale and methodology. Alzheimers Dement. (Amst) 12:e12065. 10.1002/dad2.12065 32775597 PMC7396809

[B41] Harigaya Y. Saido T. C. Eckman C. B. Prada C. M. Shoji M. Younkin S. G. (2000). Amyloid β protein starting pyroglutamate at position 3 is a major component of the amyloid deposits in the Alzheimer’s disease brain. Biochem. Biophys. Res. Commun. 276, 422–427. 10.1006/bbrc.2000.3490 11027491

[B42] Hartley S. L. Handen B. L. Devenny D. Mihaila I. Hardison R. Lao P. J.. (2017). Cognitive decline and brain amyloid-β accumulation across 3 years in adults with down syndrome. Neurobiol. Aging 58, 68–76. 10.1016/j.neurobiolaging.2017.05.019 28715661 PMC5581712

[B43] Hartley S. L. Handen B. L. Devenny D. Tudorascu D. Piro-Gambetti B. Zammit M. D.. (2020). Cognitive indicators of transition to preclinical and prodromal stages of Alzheimer’s disease in Down syndrome. Alzheimers Dement. (Amst) 12:e12096. 10.1002/dad2.12096 32995465 PMC7507534

[B44] Hartley S. L. Handen B. L. Devenny D. A. Hardison R. Mihaila I. Price J. C.. (2014). Cognitive functioning in relation to brain amyloid-β in healthy adults with Down syndrome. Brain 137, 2556–2563. 10.1093/brain/awu173 24993958 PMC4189400

[B45] He W. Barrow C. J. (1999). The A β 3-pyroglutamyl and 11-pyroglutamyl peptides found in senile plaque have greater β-sheet forming and aggregation propensities *in vitro* than full-length A β. Biochemistry 38, 10871–10877. 10.1021/bi990563r 10451383

[B46] Head E. Ances B. (2020). Biomarkers in down syndrome can help us understand Alzheimer’s disease. Lancet 395, 1951–1953. 10.1016/S0140-6736(20)30916-8 32593327

[B47] Head E. Lott I. T. Wilcock D. M. Lemere C. A. (2016). Aging in down syndrome and the development of Alzheimer’s disease neuropathology. Curr. Alzheimer Res. 13, 18–29. 10.2174/1567205012666151020114607 26651341 PMC4948181

[B48] Head E. Phelan M. J. Doran E. Kim R. C. Poon W. W. Schmitt F. A.. (2017). Cerebrovascular pathology in down syndrome and Alzheimer disease. Acta Neuropathol. Commun. 5:93. 10.1186/s40478-017-0499-4 29195510 PMC5709935

[B49] Hendrix J. A. Airey D. C. Britton A. Burke A. D. Capone G. T. Chavez R.. (2021). Cross-sectional exploration of plasma biomarkers of Alzheimer’s disease in down syndrome: early data from the longitudinal investigation for enhancing down syndrome research (LIFE-DSR) study. J. Clin. Med. 10:1907. 10.3390/jcm10091907 33924960 PMC8124643

[B50] Hosoda R. Saido T. C. Otvos L. Jr. Arai T. Mann D. M. Lee V. M.. (1998). Quantification of modified amyloid β peptides in Alzheimer disease and Down syndrome brains. J. Neuropathol. Exp. Neurol. 57, 1089–1095. 10.1097/00005072-199811000-00012 9825946

[B51] Ikonomovic M. D. Buckley C. J. Abrahamson E. E. Kofler J. K. Mathis C. A. Klunk W. E.. (2020). Post-mortem analyses of PiB and flutemetamol in diffuse and cored amyloid-β plaques in Alzheimer’s disease. Acta Neuropathol. 140, 463–476. 10.1007/s00401-020-02175-1 32772265 PMC7498488

[B52] Ikonomovic M. D. Klunk W. E. Abrahamson E. E. Mathis C. A. Price J. C. Tsopelas N. D.. (2008). Post-mortem correlates of *in vivo* PiB-PET amyloid imaging in a typical case of Alzheimer’s disease. Brain 131, 1630–1645. 10.1093/brain/awn016 18339640 PMC2408940

[B53] Iwatsubo T. Mann D. M. Odaka A. Suzuki N. Ihara Y. (1995). Amyloid β protein (A β) deposition: a β 42(43) precedes A β 40 in Down syndrome. Ann. Neurol. 37, 294–299. 10.1002/ana.410370305 7695229

[B54] Iwatsubo T. Odaka A. Suzuki N. Mizusawa H. Nukina N. Ihara Y.. (1994). Visualization of A β 42(43) and A β 40 in senile plaques with end-specific A β monoclonals: evidence that an initially deposited species is A β 42 (43). Neuron 13, 45–53. 10.1016/0896-6273(94)90458-8 8043280

[B55] Iwatsubo T. Saido T. C. Mann D. M. Lee V. M. Trojanowski J. Q. (1996). Full-length amyloid-β (1–42(43)) and amino-terminally modified and truncated amyloid-β 42(43) deposit in diffuse plaques. Am. J. Pathol. 149, 1823–1830. 8952519 PMC1865366

[B56] Jarrett J. T. Berger E. P. Lansbury P. T. Jr. (1993). The carboxy terminus of the β amyloid protein is critical for the seeding of amyloid formation: implications for the pathogenesis of Alzheimer’s disease. Biochemistry 32, 4693–4697. 10.1021/bi00069a001 8490014

[B57] Jawhar S. Wirths O. Bayer T. A. (2011). Pyroglutamate amyloid-β (Aβ): a hatchet man in Alzheimer disease. J. Biol. Chem. 286, 38825–38832. 10.1074/jbc.R111.288308 21965666 PMC3234707

[B58] Johnson K. A. Gregas M. Becker J. A. Kinnecom C. Salat D. H. Moran E. K.. (2007). Imaging of amyloid burden and distribution in cerebral amyloid angiopathy. Ann. Neurol. 62, 229–234. 10.1002/ana.21164 17683091

[B59] Jones D. T. Machulda M. M. Vemuri P. McDade E. M. Zeng G. Senjem M. L.. (2011). Age-related changes in the default mode network are more advanced in Alzheimer disease. Neurology 77, 1524–1531. 10.1212/WNL.0b013e318233b33d 21975202 PMC3198977

[B60] Kim K. S. Wen G. Y. Bancher C. Chen C. M. J. Sapienza V. J. Hong H.. (1990). Detection, quantitation of amyloid β-peptide with 2 monoclonal antibodies. Neurosci. Res. Commun. 7, 113–122.

[B61] Klunk W. E. Engler H. Nordberg A. Wang Y. Blomqvist G. Holt D. P.. (2004). Imaging brain amyloid in Alzheimer’s disease with pittsburgh compound-B. Ann. Neurol. 55, 306–319. 10.1002/ana.20009 14991808

[B62] Kuo Y. M. Emmerling M. R. Woods A. S. Cotter R. J. Roher A. E. (1997). Isolation, chemical characterization and quantitation of A β 3-pyroglutamyl peptide from neuritic plaques and vascular amyloid deposits. Biochem. Biophys. Res. Commun. 237, 188–191. 10.1006/bbrc.1997.7083 9266855

[B63] Landt J. D’Abrera J. C. Holland A. J. Aigbirhio F. I. Fryer T. D. Canales R.. (2011). Using positron emission tomography and Carbon 11-labeled pittsburgh compound B to image brain fibrillar β-amyloid in adults with down syndrome: safety, acceptability and feasibility. Arch. Neurol. 68, 890–896. 10.1001/archneurol.2011.36 21403005

[B64] Lao P. J. Betthauser T. J. Hillmer A. T. Price J. C. Klunk W. E. Mihaila I.. (2016). The effects of normal aging on amyloid-β deposition in nondemented adults with Down syndrome as imaged by carbon 11-labeled pittsburgh compound B. Alzheimers Dement. 12, 380–390. 10.1016/j.jalz.2015.05.013 26079411 PMC4677061

[B65] Lao P. J. Handen B. L. Betthauser T. J. Mihaila I. Hartley S. L. Cohen A. D.. (2018). Alzheimer-like pattern of hypometabolism emerges with elevated amyloid-β burden in down syndrome. J. Alzheimers Dis. 61, 631–644. 10.3233/JAD-170720 29254096 PMC5994924

[B66] Lao P. J. Handen B. L. Betthauser T. J. Mihaila I. Hartley S. L. Cohen A. D.. (2017). Longitudinal changes in amyloid positron emission tomography and volumetric magnetic resonance imaging in the nondemented down syndrome population. Alzheimers Dement. (Amst) 9, 1–9. 10.1016/j.dadm.2017.05.001 28603769 PMC5454131

[B67] Lemere C. A. Blusztajn J. K. Yamaguchi H. Wisniewski T. Saido T. C. Selkoe D. J.. (1996). Sequence of deposition of heterogeneous amyloid β-peptides and APOE in down syndrome: implications for initial events in amyloid plaque formation. Neurobiol. Dis. 3, 16–32. 10.1006/nbdi.1996.0003 9173910

[B68] LeVine H. 3rd Spielmann H. P. Matveev S. Cauvi F. M. Murphy M. P. Beckett T. L.. (2017). Down syndrome: age-dependence of PiB binding in postmortem frontal cortex across the lifespan. Neurobiol. Aging 54, 163–169. 10.1016/j.neurobiolaging.2017.03.005 28385551 PMC5638297

[B69] Lockhart A. Lamb J. R. Osredkar T. Sue L. I. Joyce J. N. Ye L.. (2007). PIB is a non-specific imaging marker of amyloid-β (Aβ) peptide-related cerebral amyloidosis. Brain 130, 2607–2615. 10.1093/brain/awm191 17698496

[B70] Lott I. T. Head E. (2019). Dementia in down syndrome: unique insights for Alzheimer disease research. Nat. Rev. Neurol. 15, 135–147. 10.1038/s41582-018-0132-6 30733618 PMC8061428

[B71] Ly J. V. Donnan G. A. Villemagne V. L. Zavala J. A. Ma H. O’Keefe G.. (2010). 11C-PIB binding is increased in patients with cerebral amyloid angiopathy-related hemorrhage. Neurology 74, 487–493. 10.1212/WNL.0b013e3181cef7e3 20142615

[B72] Mak E. Bickerton A. Padilla C. Walpert M. J. Annus T. Wilson L. R.. (2019a). Longitudinal trajectories of amyloid deposition, cortical thickness and tau in Down syndrome: a deep-phenotyping case report. Alzheimers Dement. (Amst) 11, 654–658. 10.1016/j.dadm.2019.04.006 31909173 PMC6939035

[B73] Mak E. Padilla C. Annus T. Wilson L. R. Hong Y. T. Fryer T. D.. (2019b). Delineating the topography of amyloid-associated cortical atrophy in Down syndrome. Neurobiol. Aging 80, 196–202. 10.1016/j.neurobiolaging.2019.02.018 31207551

[B74] Mann D. M. Esiri M. M. (1989). The pattern of acquisition of plaques and tangles in the brains of patients under 50 years of age with Down’s syndrome. J. Neurol. Sci. 89, 169–179. 10.1016/0022-510x(89)90019-1 2522541

[B75] Mann D. M. Iwatsubo T. (1996). Diffuse plaques in the cerebellum and corpus striatum in Down’s syndrome contain amyloid beta protein (A beta) only in the form of A beta 42 (43). Neurodegeneration 5, 115–120. 10.1006/neur.1996.0017 8819131

[B76] Mann D. M. (1988). Alzheimer’s disease and Down’s syndrome. Histopathology 13, 125–137. 10.1111/j.1365-2559.1988.tb02018.x 2971602

[B77] Mann D. M. A. Davidson Y. S. Robinson A. C. Allen N. Hashimoto T. Richardson A.. (2018). Patterns and severity of vascular amyloid in Alzheimer’s disease associated with duplications and missense mutations in APP gene, Down syndrome and sporadic Alzheimer’s disease. Acta Neuropathol. 136, 569–587. 10.1007/s00401-018-1866-3 29770843 PMC6132946

[B78] Masters C. L. Simms G. Weinman N. A. Multhaup G. McDonald B. L. Beyreuther K.. (1985). Amyloid plaque core protein in Alzheimer disease and Down syndrome. Proc. Natl. Acad. Sci. U S A 82, 4245–4249. 10.1073/pnas.82.12.4245 3159021 PMC397973

[B79] Mathis C. A. Lopresti B. J. Ikonomovic M. D. Klunk W. E. (2017). Small-molecule PET tracers for imaging proteinopathies. Semin. Nucl. Med. 47, 553–575. 10.1053/j.semnuclmed.2017.06.003 28826526 PMC5657567

[B80] McKhann G. Drachman D. Folstein M. Katzman R. Price D. Stadlan E. M. (1984). Clinical diagnosis of Alzheimer’s disease: report of the NINCDS-ADRDA work group under the auspices of department of health and human services task force on Alzheimer’s disease. Neurology 34, 939–944. 10.1212/wnl.34.7.939 6610841

[B81] Michno W. Nystrom S. Wehrli P. Lashley T. Brinkmalm G. Guerard L.. (2019). Pyroglutamation of amyloid-betax-42 (Abetax-42) followed by Abeta140 deposition underlies plaque polymorphism in progressing Alzheimer’s disease pathology. J. Biol. Chem. 294, 6719–6732. 10.1074/jbc.RA118.006604 30814252 PMC6497931

[B82] Mihaila I. Handen B. L. Christian B. T. Lao P. J. Cody K. A. Klunk W. E.. (2019). Leisure activity, brain beta-amyloid and episodic memory in adults with Down syndrome. Dev. Neurobiol. 79, 738–749. 10.1002/dneu.22677 30912871 PMC7063586

[B83] Miller D. L. Papayannopoulos I. A. Styles J. Bobin S. A. Lin Y. Y. Biemann K.. (1993). Peptide compositions of the cerebrovascular and senile plaque core amyloid deposits of Alzheimer’s disease. Arch. Biochem. Biophys. 301, 41–52. 10.1006/abbi.1993.1112 8442665

[B84] Mintun M. A. Lo A. C. Duggan Evans C. Wessels A. M. Ardayfio P. A. Andersen S. W.. (2021). Donanemab in early Alzheimer’s disease. N. Engl. J. Med. 384, 1691–1704. 10.1056/NEJMoa2100708 33720637

[B85] Mirra S. S. Heyman A. McKeel D. Sumi S. M. Crain B. J. Brownlee L. M.. (1991). The consortium to establish a registry for Alzheimer’s disease (CERAD). part II. standardization of the neuropathologic assessment of Alzheimer’s disease. Neurology 41, 479–486. 10.1212/wnl.41.4.479 2011243

[B86] Montine T. J. Phelps C. H. Beach T. G. Bigio E. H. Cairns N. J. Dickson D. W.. (2012). National institute on aging-Alzheimer’s association guidelines for the neuropathologic assessment of Alzheimer’s disease: a practical approach. Acta Neuropathol. 123, 1–11. 10.1007/s00401-011-0910-3 22101365 PMC3268003

[B87] Morawski M. Schilling S. Kreuzberger M. Waniek A. Jager C. Koch B.. (2104). Glutaminyl cyclase in human cortex: correlation with (pGlu)-amyloid-beta load and cognitive decline in Alzheimer’s disease. J. Alzheimers Dis. 39, 385–400. 10.3233/JAD-131535 24164736

[B88] Mori C. Spooner E. T. Wisniewsk K. E. Wisniewski T. M. Yamaguch H. Saido T. C.. (2002). Intraneuronal Abeta42 accumulation in Down syndrome brain. Amyloid 9, 88–102. 10.3109/13506120208995241 12440481

[B89] Murdoch J. C. Rodger J. C. Rao S. S. Fletcher C. D. Dunnigan M. G. (1977). Down’s syndrome: an atheroma-free model. Br. Med. J. 2, 226–228. 141966 10.1136/bmj.2.6081.226 PMC1631400

[B90] Murray M. E. Lowe V. J. Graff-Radford N. R. Liesinger A. M. Cannon A. Przybelski S. A.. (2015). Clinicopathologic and 11C-Pittsburgh compound B implications of Thal amyloid phase across the Alzheimer’s disease spectrum. Brain 138, 1370–1381. 10.1093/brain/awv050 25805643 PMC4407190

[B91] Neale N. Padilla C. Fonseca L. M. Holland T. Zaman S. (2018). Neuroimaging and other modalities to assess Alzheimer’s disease in Down syndrome. Neuroimage Clin. 17, 263–271. 10.1016/j.nicl.2017.10.022 29159043 PMC5683343

[B92] Olichney J. M. Hansen L. A. Hofstetter C. R. Grundman M. Katzman R. Thal L. J.. (1995). Cerebral infarction in Alzheimer’s disease is associated with severe amyloid angiopathy and hypertension. Arch. Neurol. 52, 702–708. 10.1001/archneur.1995.00540310076019 7619027

[B93] Oyama F. Cairns N. J. Shimada H. Oyama R. Titani K. Ihara Y.. (1994). Down’s syndrome: up-regulation of beta-amyloid protein precursor and tau mRNAs and their defective coordination. J. Neurochem. 62, 1062–1066. 10.1046/j.1471-4159.1994.62031062.x 8113792

[B94] Palmqvist S. Scholl M. Strandberg O. Mattsson N. Stomrud E. Zetterberg H.. (2017). Earliest accumulation of beta-amyloid occurs within the default-mode network and concurrently affects brain connectivity. Nat. Commun. 8:1214. 10.1038/s41467-017-01150-x 29089479 PMC5663717

[B95] Perez S. E. Miguel J. C. He B. Malek-Ahmadi M. Abrahamson E. E. Ikonomovic M. D.. (2019). Frontal cortex and striatal cellular and molecular pathobiology in individuals with Down syndrome with and without dementia. Acta Neuropathol. 137, 413–436. 10.1007/s00401-019-01965-6 30734106 PMC6541490

[B96] Petersen M. E. Rafii M. S. Zhang F. Hall J. Julovich D. Ances B. M.. (2021). plasma total-tau and neurofilament light chain as diagnostic biomarkers of Alzheimer’s disease dementia and mild cognitive impairment in adults with Down syndrome. J. Alzheimers Dis. 79, 671–681. 10.3233/JAD-201167 33337378 PMC8273927

[B97] Petersen M. E. Zhang F. Schupf N. Krinsky-McHale S. J. Hall J. Mapstone M.. (2020). Proteomic profiles for Alzheimer’s disease and mild cognitive impairment among adults with Down syndrome spanning serum and plasma: an Alzheimer’s biomarker consortium-Down syndrome (ABC-DS) study. Alzheimers Dement. (Amst) 12:e12039. 10.1002/dad2.12039 32626817 PMC7327223

[B98] Pfeifer L. A. White L. R. Ross G. W. Petrovitch H. Launer L. J. (2002). Cerebral amyloid angiopathy and cognitive function: the HAAS autopsy study. Neurology 58, 1629–1634. 10.1212/wnl.58.11.1629 12058090

[B99] Pivtoraiko V. N. Abrahamson E. E. Leurgans S. E. DeKosky S. T. Mufson E. J. Ikonomovic M. D.. (2015). Cortical pyroglutamate amyloid-beta levels and cognitive decline in Alzheimer’s disease. Neurobiol. Aging. 36, 12–19. 10.1016/j.neurobiolaging.2014.06.021 25048160 PMC4268150

[B100] Planton M. Saint-Aubert L. Raposo N. Payoux P. Salabert A. S. Albucher J. F.. (2020). Florbetapir regional distribution in cerebral amyloid angiopathy and Alzheimer’s disease: a PET study. J. Alzheimers Dis. 73, 1607–1614. 10.3233/JAD-190625 31958082 PMC7081105

[B101] Provenzano F. A. Muraskin J. Tosto G. Narkhede A. Wasserman B. T. Griffith E. Y.. (2013). White matter hyperintensities and cerebral amyloidosis: necessary and sufficient for clinical expression of Alzheimer disease. JAMA Neurol. 70, 455–461. 10.1001/jamaneurol.2013.1321 23420027 PMC4124641

[B102] Pucci F. Machado G. Solera E. Cenovicz F. Arruda C. Braga C.. (2016). Blood pressure levels and body mass index in brazilian adults with Down syndrome. Sao Paulo Med. J. 134, 330–334. 10.1590/1516-3180.2016.0057180316 27557142 PMC10876337

[B103] Rafii M. S. Ances B. M. Schupf N. Krinsky-McHale S. J. Mapstone M. Silverman W.. (2020). The AT(N) framework for Alzheimer’s disease in adults with Down syndrome. Alzheimers Dement. (Amst) 12:e12062. 10.1002/dad2.12062 33134477 PMC7588820

[B104] Roher A. E. Kokjohn T. A. Clarke S. G. Sierks M. R. Maarouf C. L. Serrano G. E.. (2017). APP/Abeta structural diversity and Alzheimer’s disease pathogenesis. Neurochem. Int. 110, 1–13. 10.1016/j.neuint.2017.08.007 28811267 PMC5688956

[B105] Roher A. E. Lowenson J. D. Clarke S. Woods A. S. Cotter R. J. Gowing E.. (1993). beta-Amyloid-(1–42) is a major component of cerebrovascular amyloid deposits: implications for the pathology of Alzheimer disease. Proc. Natl. Acad. Sci. U S A 90, 10836–10840. 10.1073/pnas.90.22.10836 8248178 PMC47873

[B106] Roher A. E. Palmer K. C. Yurewicz E. C. Ball M. J. Greenberg B. D. (1993). Morphological and biochemical analyses of amyloid plaque core proteins purified from Alzheimer disease brain tissue. J. Neurochem. 61, 1916–1926. 10.1111/j.1471-4159.1993.tb09834.x 8229002

[B107] Rowe C. C. Ng S. Ackermann U. Gong S. J. Pike K. Savage G.. (2007). Imaging beta-amyloid burden in aging and dementia. Neurology 68, 1718–1725. 10.1212/01.wnl.0000261919.22630.ea 17502554

[B109] Russo C. Saido T. C. DeBusk L. M. Tabaton M. Gambetti P. Teller J. K.. (1997). Heterogeneity of water-soluble amyloid beta-peptide in Alzheimer’s disease and Down’s syndrome brains. FEBS Lett. 409, 411–416. 10.1016/s0014-5793(97)00564-4 9224700

[B110] Sabbagh M. N. Fleisher A. Chen K. Rogers J. Berk C. Reiman E.. (2011). Positron emission tomography and neuropathologic estimates of fibrillar amyloid-beta in a patient with Down syndrome and Alzheimer disease. Arch. Neurol. 68, 1461–1466. 10.1001/archneurol.2011.535 22084131 PMC3346179

[B111] Saido T. C. Iwatsubo T. Mann D. M. Shimada H. Ihara Y. Kawashima S.. (1995). Dominant and differential deposition of distinct beta-amyloid peptide species, A beta N3(pE), in senile plaques. Neuron 14, 457–466. 10.1016/0896-6273(95)90301-1 7857653

[B112] Saido T. C. Yamao-Harigaya W. Iwatsubo T. Kawashima S. (1996). Amino- and carboxyl-terminal heterogeneity of beta-amyloid peptides deposited in human brain. Neurosci. Lett. 215, 173–176. 10.1016/0304-3940(96)12970-0 8899741

[B113] Schilling S. Lauber T. Schaupp M. Manhart S. Scheel E. Bohm G.. (2006). On the seeding and oligomerization of pGlu-amyloid peptides *(in vitro)*. Biochemistry 45, 12393–12399. 10.1021/bi0612667 17029395

[B114] Schilling S. Zeitschel U. Hoffmann T. Heiser U. Francke M. Kehlen A.. (2008). Glutaminyl cyclase inhibition attenuates pyroglutamate Abeta and Alzheimer ’s disease-like pathology. Nat. Med. 14, 1106–1111. 10.1038/nm.1872 18836460

[B115] Schlenzig D. Manhart S. Cinar Y. Kleinschmidt M. Hause G. Willbold D.. (2009). Pyroglutamate formation influences solubility and amyloidogenicity of amyloid peptides. Biochemistry 48, 7072–7078. 10.1021/bi900818a 19518051

[B116] Seo S. W. Ayakta N. Grinberg L. T. Villeneuve S. Lehmann M. Reed B.. (2017). Regional correlations between [(11)C]PIB PET and post-mortem burden of amyloid-beta pathology in a diverse neuropathological cohort. Neuroimage Clin. 13, 130–137. 10.1016/j.nicl.2016.11.008 27981028 PMC5144753

[B117] Sullivan C. P. Berg E. A. Elliott-Bryant R. Fishman J. B. McKee A. C. Morin P. J.. (2011). Pyroglutamate-Abeta 3 and 11 colocalize in amyloid plaques in Alzheimer’s disease cerebral cortex with pyroglutamate-Abeta 11 forming the central core. Neurosci. Lett. 505, 109–112. 10.1016/j.neulet.2011.09.071 22001577 PMC3253715

[B118] Teller J. K. Russo C. DeBusk L. M. Angelini G. Zaccheo D. Dagna-Bricarelli F.. (1996). Presence of soluble amyloid beta-peptide precedes amyloid plaque formation in Down’s syndrome. Nat. Med. 2, 93–95. 10.1038/nm0196-93 8564851

[B119] Tudorascu D. L. Anderson S. J. Minhas D. S. Yu Z. Comer D. Lao P.. (2019). Comparison of longitudinal Abeta in nondemented elderly and Down syndrome. Neurobiol. Aging. 73, 171–176. 10.1016/j.neurobiolaging.2018.09.030 30359879 PMC6251757

[B120] Tudorascu D. L. Laymon C. M. Zammit M. Minhas D. S. Anderson S. J. Ellison P. A.. (2020). Relationship of amyloid beta and neurofibrillary tau deposition in neurodegeneration in aging Down syndrome (NiAD) study at baseline. Alzheimers Dement. (N Y) 6:e12096. 10.1002/trc2.12096 33163613 PMC7602678

[B121] Villemagne V. L. Barkhof F. Garibotto V. Landau S. M. Nordberg A. van Berckel B. N. M.. (2021). Molecular imaging approaches in dementia. Radiology 298, 517–530. 10.1148/radiol.2020200028 33464184 PMC7924525

[B122] Vinters H. V. (1987). Cerebral amyloid angiopathy. A critical review. Stroke 18, 311–324. 10.1161/01.str.18.2.311 3551211

[B123] Viswanathan A. Greenberg S. M. (2011). Cerebral amyloid angiopathy in the elderly. Ann. Neurol. 70, 871–880. 10.1002/ana.22516 22190361 PMC4004372

[B124] Wilcock D. M. Schmitt F. A. Head E. (2016). Cerebrovascular contributions to aging and Alzheimer’s disease in Down syndrome. Biochim. Biophys Acta. 1862, 909–914. 10.1016/j.bbadis.2015.11.007 26593849 PMC4821721

[B125] Wilson L. R. Vatansever D. Annus T. Williams G. B. Hong Y. T. Fryer T. D.. (2019). Differential effects of Down’s syndrome and Alzheimer ’s neuropathology on default mode connectivity. Hum. Brain Mapp. 40, 4551–4563. 10.1002/hbm.24720 31350817 PMC6865660

[B126] Wisniewski K. E. Wisniewski H. M. Wen G. Y. (1985). Occurrence of neuropathological changes and dementia of Alzheimer’s disease in Down’s syndrome. Ann. Neurol. 17, 278–282. 10.1002/ana.410170310 3158266

[B127] Yamada M. (2015). Cerebral amyloid angiopathy: emerging concepts. J. Stroke 17, 17–30. 10.5853/jos.2015.17.1.17 25692104 PMC4325636

[B128] Yamin G. Teplow D. B. (2017). Pittsburgh compound-B (PiB) binds amyloid beta-protein protofibrils. J. Neurochem. 140, 210–215. 10.1111/jnc.13887 27943341 PMC5225051

[B129] Zammit M. D. Laymon C. M. Betthauser T. J. Cody K. A. Tudorascu D. L. Minhas D. S.. (2020). Amyloid accumulation in Down syndrome measured with amyloid load. Alzheimers Dement. (Amst) 12:e12020. 10.1002/dad2.12020 32435686 PMC7233422

[B130] Zammit M. D. Tudorascu D. L. Laymon C. M. Hartley S. L. Zaman S. H. Ances B. M.. (2021). PET measurement of longitudinal amyloid load identifies the earliest stages of amyloid-beta accumulation during Alzheimer’s disease progression in Down syndrome. Neuroimage 228:117728. 10.1016/j.neuroimage.2021.117728 33421595 PMC7953340

[B131] Zigman W. B. Devenny D. A. Krinsky-McHale S. J. Jenkins E. C. Urv T. K. Wegiel J.. (2008). Alzheimer’s disease in adults with Down syndrome. Int. Rev. Res. Ment. Retard. 36, 103–145. 10.1016/S0074-7750(08)00004-9 19633729 PMC2714652

[B132] Zigman W. B. Schupf N. Urv T. Zigman A. Silverman W. (2002). Incidence and temporal patterns of adaptive behavior change in adults with mental retardation. Am. J. Ment. Retard. 107, 161–174. 10.1352/0895-8017(2002)107<0161:IATPOA>2.0.CO;2 11966329

